# Investigation of the *Cyanothece* nitrogenase cluster in *Synechocystis*: a blueprint for engineering nitrogen-fixing photoautotrophs

**DOI:** 10.1128/mbio.04052-24

**Published:** 2025-02-25

**Authors:** Deng Liu, Anindita Bandyopadhyay, Michelle Liberton, Himadri B. Pakrasi, Maitrayee Bhattacharyya-Pakrasi

**Affiliations:** 1 Department of Biology, Washington University, St. Louis, Missouri, USA; University of Washington School of Medicine, Seattle, Washington, USA

**Keywords:** nitrogen fixation, cyanobacteria, reconstruction, essential *nif *genes

## Abstract

The nitrogenase gene cluster of unicellular diazotrophic cyanobacteria, such as *Cyanothece*, is frequently selected by nature for nitrogen-fixing partnerships with eukaryotic phototrophs. The essential cluster components that confer an advantage in such partnerships remain underexplored. To use this cluster for the development of synthetic, phototrophic nitrogen-fixing systems, a thorough and systematic analysis of its constituent genes is necessary. An initial effort to assess the possibility of engineering this cluster into non-diazotrophic phototrophs led to the generation of a *Synechocystis* 6803 strain with significant nitrogenase activity. In the current study, a refactoring approach was taken to determine the dispensability of the non-structural genes in the cluster and define a minimal gene set for constructing a functional nitrogenase for phototrophs. Using a bottom-up strategy, the *nif* genes from *Cyanothece* 51142 were re-organized to form new operons. The genes were then seamlessly removed to determine their essentiality in the nitrogen fixation process. We demonstrate that besides the structural genes *nifHDK*, *nifBSUENPVZTXW*, as well as *hesAB*, are important for optimal nitrogenase function in a phototroph. We also show that optimal expression of these genes is crucial for efficient nitrogenase activity. Our findings provide a solid foundation for generating synthetic systems that will facilitate solar-powered conversion of atmospheric nitrogen into nitrogen-rich compounds, a stride toward a greener world.

**IMPORTANCE:**

Integrating nitrogen fixation genes into various photosynthetic organisms is an exciting strategy for converting atmospheric nitrogen into nitrogen-rich products in a green and energy-efficient way. In order to facilitate this process, it is essential that we understand the fundamentals of the functioning of a prokaryotic nitrogen-fixing machinery in a non-diazotrophic, photoautotrophic cell. This study examines a nitrogenase gene cluster that has been naturally selected on multiple occasions for a nitrogen-fixing partnership by eukaryotic photoautotrophs and provides a basic blueprint for designing a photosynthetic organism with nitrogen-fixing ability.

## INTRODUCTION

Multiple examples of natural endosymbiotic events suggest a propensity for the nitrogenase (*nif*) gene cluster of unicellular cyanobacteria of the order Chroococcales to be selected for photosynthetic–diazotrophic partnerships. Of these, the *Cyanothece nif* cluster has been referred to as a ‘privileged’ partner, forming associations with diatoms, such as *Rhopalodia*, *Epithemia*, and *Climacodium*, as well as the coccolithophore *Braarudosphaera bigelowii* (1–5). These naturally occurring instances suggest that the *Cyanothece nif* cluster harbors features that are conducive for nitrogenase to function in a photoautotroph and demands a thorough investigation as we intensify our efforts toward developing synthetic nitrogen- and carbon-fixing systems. In a first-of-its kind effort in this direction, the unicellular non-diazotrophic cyanobacterium *Synechocystis* sp. PCC 6803 (hereafter *Synechocystis* 6803) was engineered to fix nitrogen by introduction of the nitrogenase gene cluster from *Cyanothece* sp. ATCC 51142 (6). The resulting strain exhibited significant rates of nitrogen fixation, affirming the potential of this cluster to generate functional nitrogen-fixing machinery in photoautotrophs and initiating our next round of efforts into its more in-depth analysis.

The commonly occurring nitrogenase enzyme is composed of two metalloproteins: the MoFe protein (dinitrogenase, NifDK) and the Fe protein (dinitrogenase reductase, NifH) (7). Besides these structural subunits of the enzyme, three iron–sulfur clusters (F, *P*, and M clusters) are required to form a mature nitrogenase enzyme complex (8, 9). The processes of assembly and insertion of these three iron–sulfur clusters into the nitrogenase enzyme are complicated, involving multiple steps (10). The genetic elements required for the nitrogenase enzyme to mature vary in different diazotrophic species (11, 12). Previous efforts to engineer *Escherichia coli* with nitrogen fixation experimented with various combinations of *nif* genes as a strategy to optimize the activity. The study identified nine genes from the *nif* cluster of *Paenibacillus* sp. WLY78, including *nifHDK* and *nifBENXhesAnifV*, as essential (13). A different study determined 10 genes (*anfHDGK* and *nifJUSVFB*) from the *nif* clusters of *Azotobacter vinelandii* and *Klebsiella oxytoca* to be essential for nitrogenase activity (14). On the contrary, a recent report by Yang et al. identified 13 genes, including *nifHDK* and *nifENBYUSVFMJ*, as the essential components of the nitrogenase cluster of *Klebsiella oxytoca* (15). Nitrogenase activity could be further improved 10-fold using a refactoring approach and expressing 18 *nif* genes in various combinations, along with additional genes, and by altering the expression ratios of genes (16).

In our earlier work, we demonstrated the feasibility of expressing the native 24 gene *nif* cluster from *Cyanothece* 51142 in *Synechocystis* 6803. The additional seven genes in the cluster were not included in this study (6). Furthermore, except for the structural genes *nifHDK*, the essentiality of the other genes in the cluster in nitrogen fixation was not determined in this photosynthetic chassis. In the current study, we reconstructed *nif* gene operons using a bottom–up strategy in *Synechocystis* 6803. Promoters, ribosome binding sites (RBS), and transcriptional terminators previously identified (17) were used to control the expression of the *nif* genes. We found that 16 genes organized into five engineered operons formed a functional nitrogenase enzyme complex with remarkable activity. More importantly, using the CRISPR/cpf1 strategy (18), we strategically removed each gene, except *nifHDK*, from the reconstructed *nif* operons to determine their essentiality. Our study showed that deletion of any of these genes leads to a decrease in nitrogenase activity, thus establishing the minimal gene set required for optimal nitrogenase function in a non-diazotrophic photoautotroph.

## RESULTS

### Expression levels of structural genes affect nitrogenase activity

**Fig 1:**
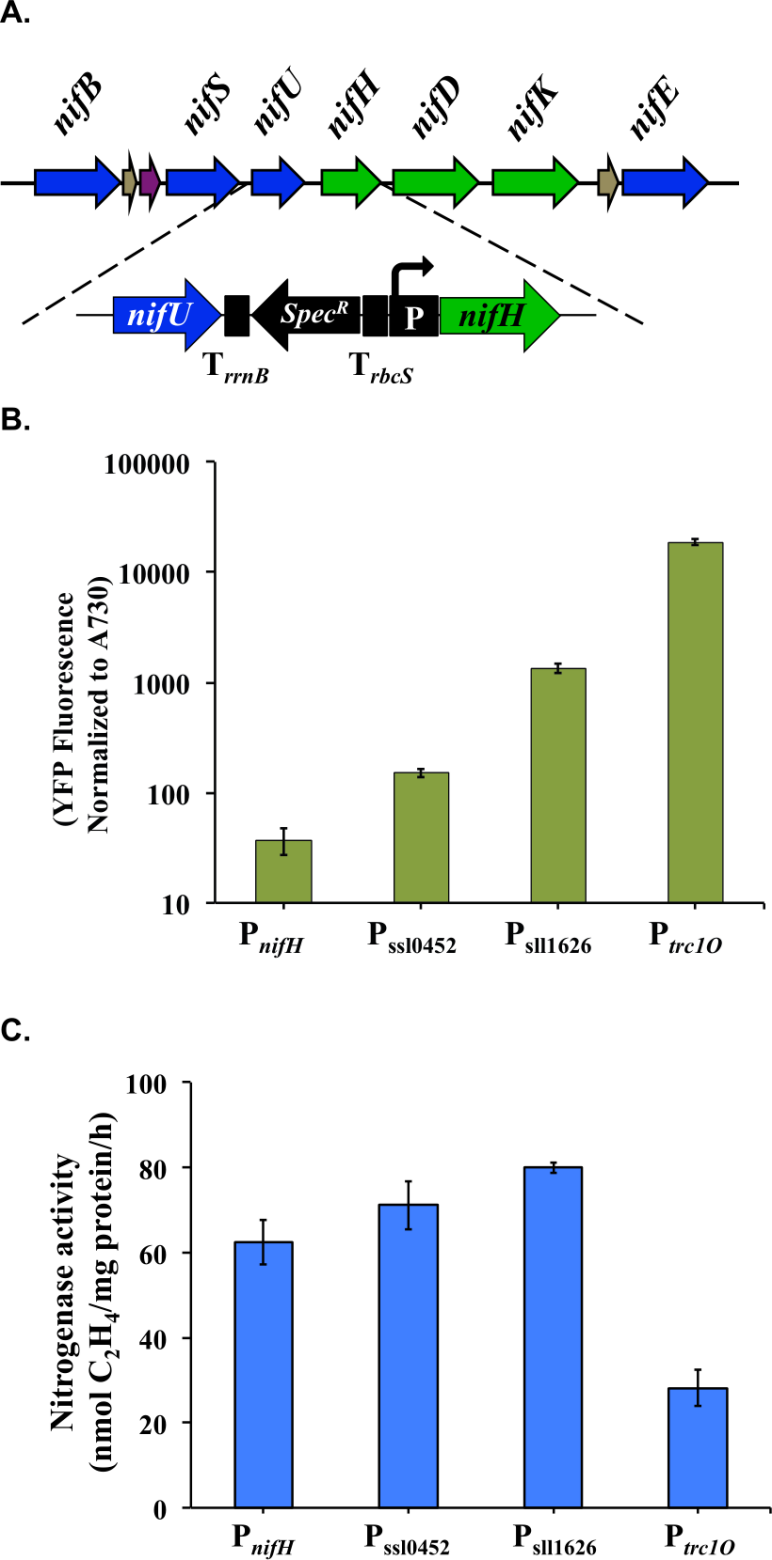
Expression levels of nitrogenase structural genes affect nitrogen fixation activities in engineered *Synechocystis* 6803. (**A**) Schematic map of changes on the 5′-UTR sequence before the *nifH* gene. Three promoters were tested, P_ssl0452_, P_sll1626_, and P*_trc1O_*. (**B**) The strengths of the tested promoters were compared to the native promoter P*_nifH_*. The strengths were tested from the expression levels of the reporter protein EYFP. (**C**) Nitrogenase activity under different strength promoters preceding the *nifH* gene. Samples were collected from cultures under 12 h light/12 h dark conditions in BG11_0_ medium. Nitrogen fixation activity was assayed by acetylene reduction, and error bars represent the standard deviations observed from at least three independent experiments.

In the *nif* cluster of *Cyanothece* 51142, the transcriptional start sites and operons are not well defined. The only well-characterized operon in this cluster is the *nifHDK* operon, the expression of which is driven by the sequence before the *nifH* gene (19). In order to test the effect of the expression strength of these genes on nitrogenase activity, we cloned the sequence between *nifU* and *nifH* into cassettes containing different promoters (Fig. 1A).

Three promoters characterized in an earlier study (17), P_sll0452_, P_sll1626_, and P*_trc1O_*, were tested for their efficacy in driving gene expression in comparison to the natural promoter P*_nifH_*. Using EYFP as the reporter protein (Fig. S1), we found that the strength of P*_nifH_* was the lowest among all the promoters tested (Fig. 1B). Nitrogenase activities could be enhanced when the stronger promoters P_sll0452_ and P_sll1626_ were used. However, when the strongest promoter P*_trc1O_* was used to drive the expression of *nifHDK*, more than 50% reduction in nitrogenase activity was observed (Fig. 1C). These results suggested that optimal expression of the structural *nif* genes is important for achieving high nitrogenase activity, a finding in accordance with a study on *Klebsiella* (20).

### Bottom–up strategy to re-organize nitrogen fixation genes

**Fig 2:**
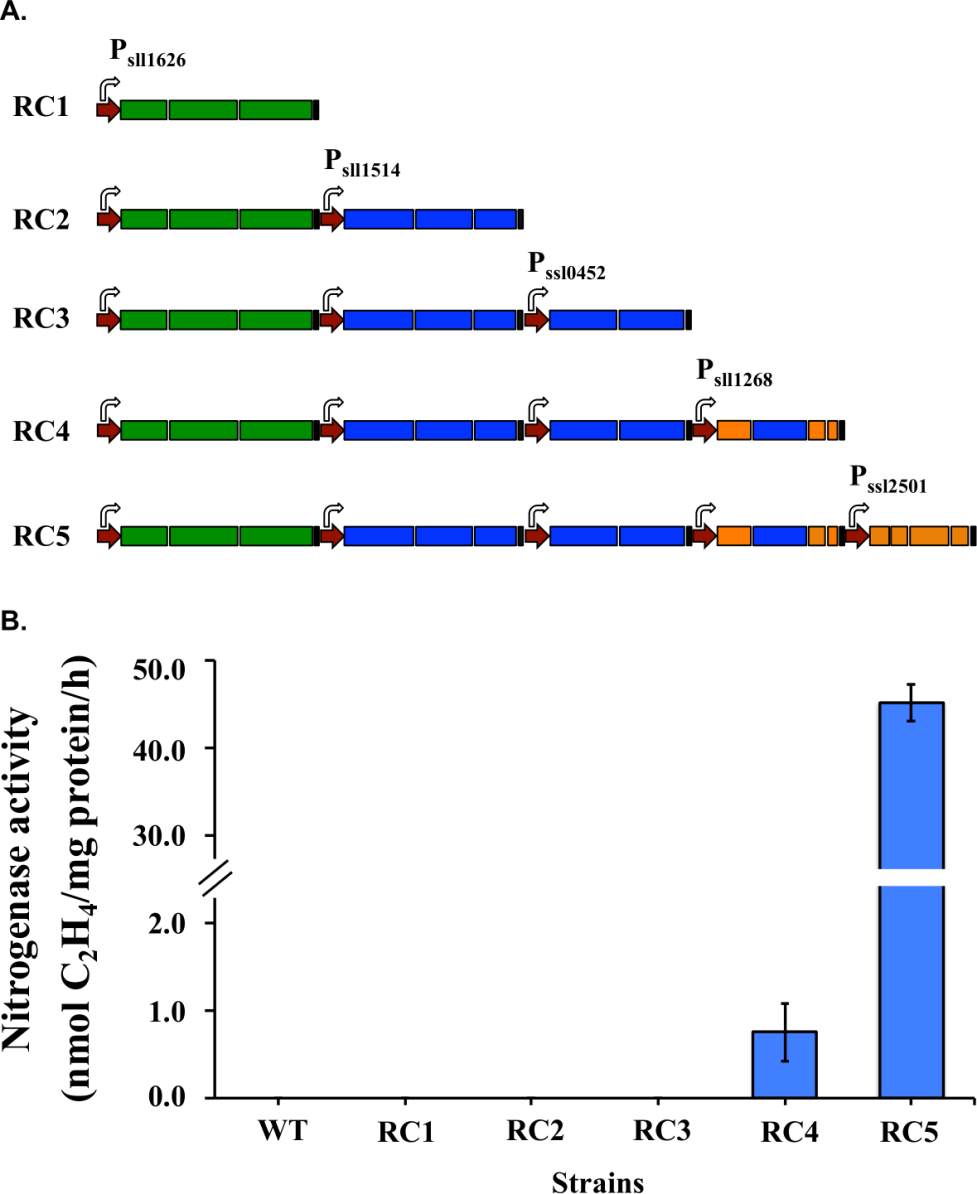
Rebuilding the functional nitrogenase enzyme in *Synechocystis* 6803. (**A**) A schematic map of reconstruction of *nif* genes using a bottom–up strategy. Five operons were added sequentially into the plasmid pCB-SC101, which were transferred into *Synechocystis* 6803 as the CK strain. For each operon, promoters, RBS, and terminators were organized with ORFs to form an independent expression cassette. Shown are promoters (red), transcription terminators (black), and the genes for the three structural proteins, *nifHDK* (green), necessary cofactors (blue), accessory proteins (orange). (**B**) Nitrogenase activities of engineered strains containing sets of reconstructed *nif* operons. Samples were collected from cultures under 12 h light/12 h dark conditions in BG11_0_ medium. Nitrogen fixation activity was assayed by acetylene reduction, and error bars represent the standard deviations observed from at least three independent experiments.

To find the essential genes for nitrogenase activity, we used a bottom–up strategy to build operons containing the genes from the native *Cyanothece* 51142 *nif* cluster (Fig. 2A). Promoters, RBS, and transcriptional terminators used in this study are native genetic elements of *Synechocystis* 6803 that were characterized earlier (17). The re-organized operons were inserted into the backbone of the shuttle vector pCB-SC101 (17).

Of the 24 genes in the native *Cyanothece nif* cluster, 16 genes have been annotated as *nif* genes in the JGI/IMG microbial database (genome.jgi.doe.gov) (21). The others are mostly genes of unknown function, except one that is annotated as a ferredoxin gene. The 16 annotated genes were sequentially organized into five independent operons, *nifHDK*, *nifBSU*, *nifEN*, *nifPVZT*, and *nifXWhesAB* (Fig. S2). Five engineered strains (RC1 to RC5) were generated by transforming *Synechocystis* 6803 with plasmids containing the above operons in different combinations. The strain RC4 containing 12 genes (*nifHDKBSUENPVZT*) showed significant activity, generating 0.75 nmol ethylene per mg total protein in 1 h(Fig. 2B). Complementing these 12 genes with the *nifXWhesAB* operon generated the RC5 strain, which exhibited a 60-fold increase in activity, thereby confirming these genes as essential components of the nitrogen-fixing machinery. This observation was in accordance with our earlier findings (6), and the RC5 strain was selected for further analyses of the cluster.

### Influences of ferredoxins on nitrogenase activity

**Fig 3:**
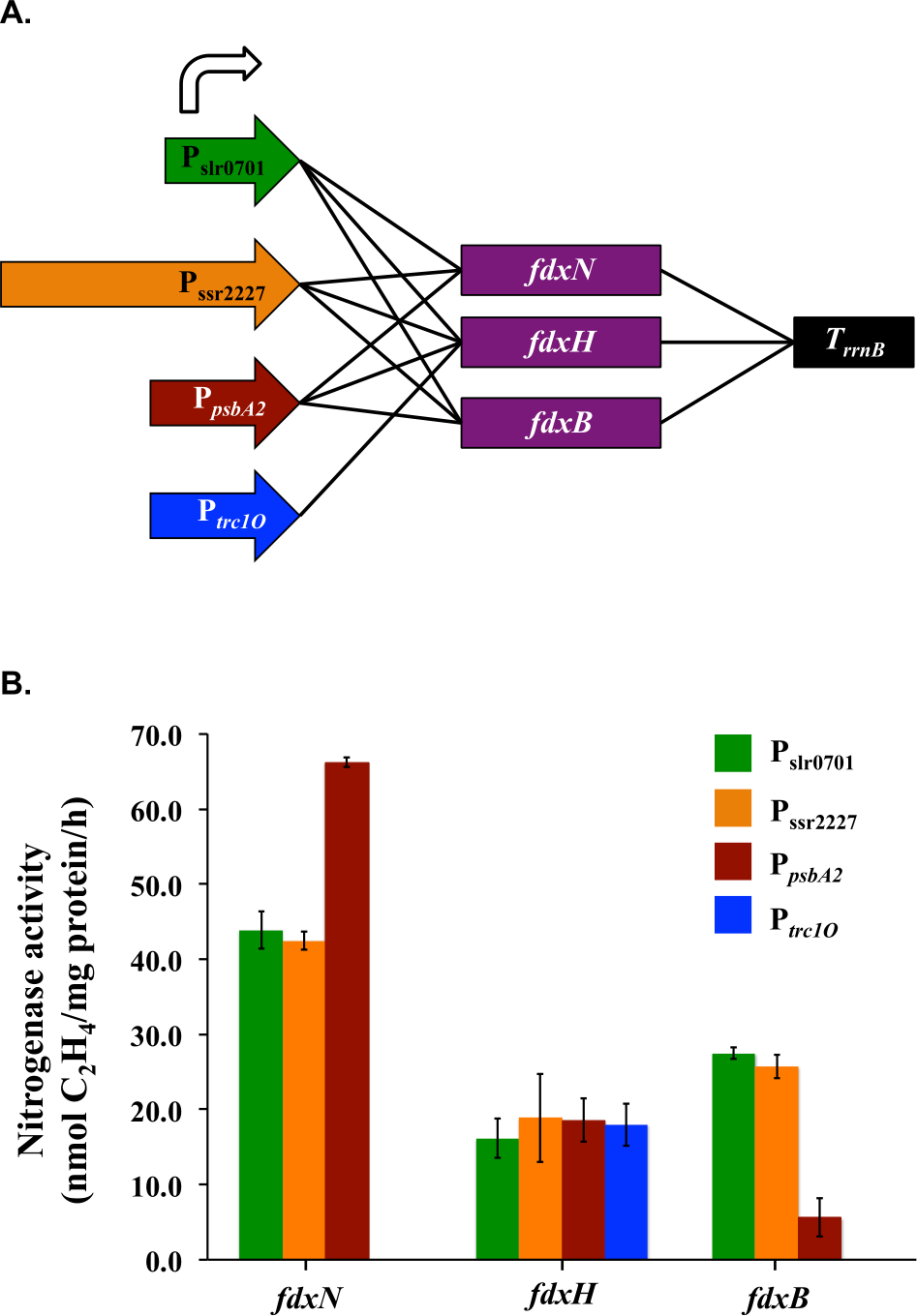
Ferredoxins affect nitrogenase activity in engineered *Synechocystis* 6803. (**A**) Scheme showing the combination of promoters and ferredoxin genes. The expression cassettes were integrated into the plasmid pRSF1010. (**B**) Nitrogenase activities are affected by type and expression level of ferredoxins in engineered *Synechocystis* 6803. Samples were collected from cultures under 12 h light/12 h dark conditions in BG11_0_ medium. Nitrogen fixation activity was assayed by acetylene reduction, and error bars represent the standard deviations observed from at least three independent experiments.

As direct electron donors to Fe protein, ferredoxins are known to significantly affect nitrogenase activity (22, 23). There is one ferredoxin annotated as *fdxN* in the 24-gene native *nif* cluster of *Cyanothece* 51142 and two more ferredoxins, *fdxH* and *fdxB*, in the 35-gene extended cluster. To evaluate the role of ferredoxins in nitrogen fixation, the three ferredoxin genes, *fdxN*, *fdxH*, and *fdxB*, were introduced into the RC5 strain. Four promoters, P_slr0701_, P_ssr2227_, P*_psbA2_*, and P*_trc1O_* were used to independently drive the expression of each ferredoxin gene (Fig. 3A). One interesting observation was that, when driven by the P*_trc1O_* promoter, only the *fdxH* gene could be transformed into *Synechocystis*. Therefore*,* for genes *fdxN* and *fdxB*, only three promoters were tested.

The ferredoxins tested had a diverse effect on nitrogenase activity (Fig. 3B). When the *fdxN* gene under the P*_psbA2_* promoter was introduced into RC5, a 50% increase in nitrogenase activity was observed. In contrast, when *fdxB* under a strong promoter was introduced into RC5, a significant reduction in activity was observed. Interestingly, the introduction of *fdxH* into RC5 leads to a 60% decrease in activity, irrespective of the promoter used. This implied that the *fdxH* gene product negatively impacts nitrogen fixation.

### Essential *nif* genes for nitrogenase activity in *Synechocystis* 6803

**Fig 4:**
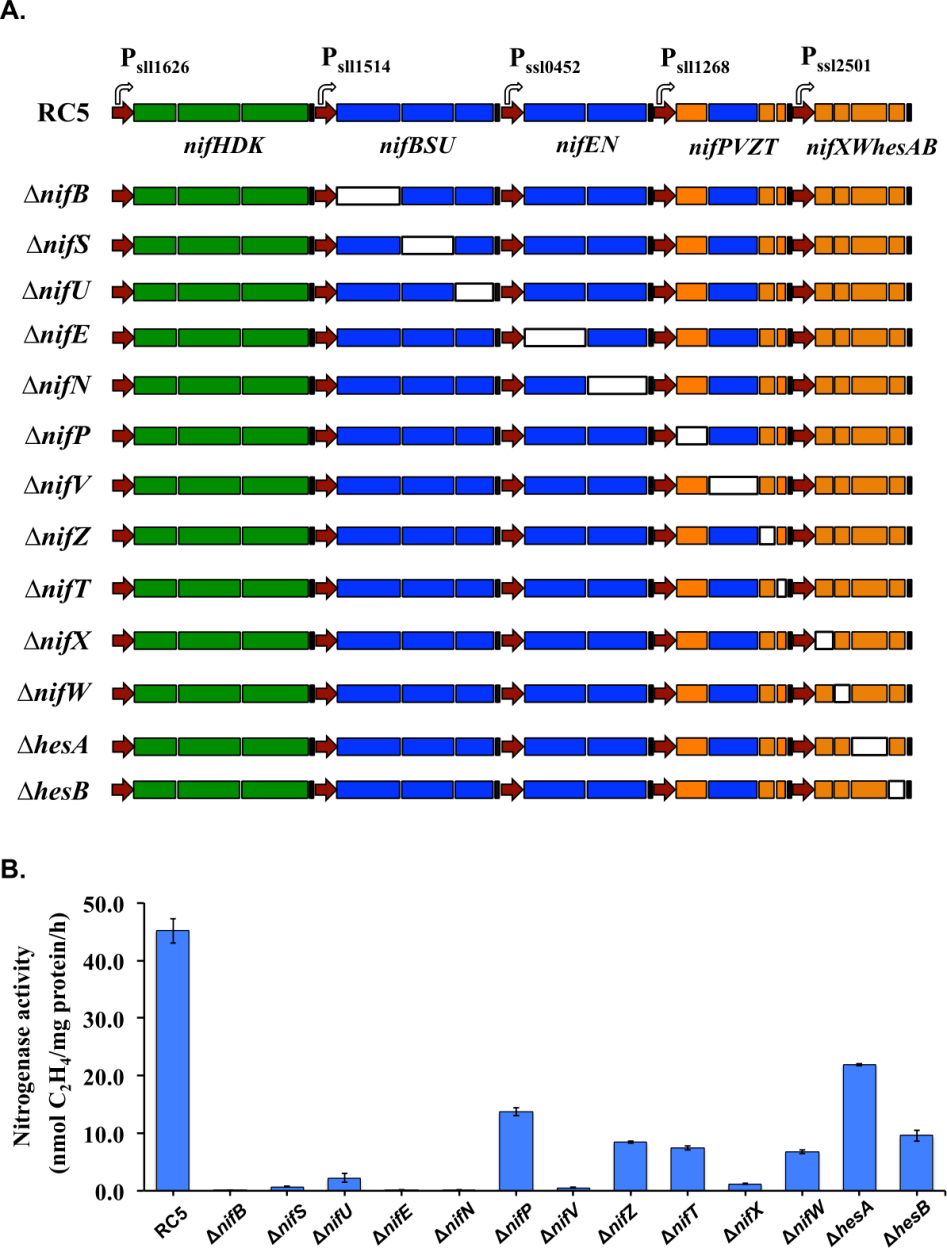
Deletion analysis of the genes shown in the re-constructed *nif* operons. (**A**) Scheme showing the deletion of specific genes in the operons using the CRISPR/cpf1 system. The hollow rectangles represent the deleted genes, and the colored rectangles represent the remaining genes. (**B**) Nitrogenase activity of the deletion strains in engineered *Synechocystis* 6803. Samples were collected from cultures under 12 h light/12 h dark conditions in a BG11_0_ medium. Nitrogen fixation activity was assayed by acetylene reduction, and error bars represent the standard deviations observed from at least three independent experiments.

Of the 16 annotated genes in strain RC5, only the structural genes *nifHDK* have been functionally characterized. The role of the other 13 genes in nitrogen fixation remains largely unknown. To determine the essential set of genes for nitrogenase activity in *Synechocystis* 6803, we used the CRISPR/cpf1 strategy (18) to seamlessly delete each of the 13 genes after the *nifHDK* operon (Fig. S3). The 13 mutant strains based on RC5 were generated as shown in Fig. 4A.

As anticipated, deletion of *nifB*, *nifE*, or *nifN* leads to a complete cessation of nitrogenase activity (Fig. 4B). This observation is in agreement with the fact that *nifENB* genes are required for M cluster biosynthesis, and they are the most conserved genes besides *nifHDK* among all diazotrophs (11, 24). Similarly, the deletion of any of the three genes *nifSU* and *nifV* resulted in over 100-fold decrease in nitrogenase activity. NifSU proteins are required for synthesis of iron–sulfur clusters (25), while *nifV* encodes the enzyme homocitrate synthase. Homocitrate is a component of the M cluster present in the catalytic center of dinitrogenase (26). NifP encodes a serine acetyltransferase, which has been identified as a key enzyme in cysteine biosynthesis in *E. coli* (27). Deletion of *nifP* (*cysE2*) resulted in approximately 60% reduction in nitrogenase activity in *Synechocystis*, implying its essential role in the process. Genes *nifZTW* encode proteins that are suggested to be involved in MoFe protein maturation, but none were found to be essential for nitrogen fixation in *E. coli* (13–15). However, our results showed that deletion of *nifZ* leads to 70% decrease in nitrogenase activity, while deletions of *nifT* and W lead to about 80% decrease, implying their essential roles in nitrogen fixation in *a* photoautotroph. NifX is an interesting protein that has been identified as having various roles in different diazotrophs. It is a negative regulator of nitrogenase activity in *Klebsiella* (28), while in *Azotobacter*, it is required for the biosynthesis of the M cluster (29). Deletion of *nifX* in *Synechocystis* 6803 resulted in an over 100-fold decrease in nitrogenase activity, implying its essential role. Genes *hesAB* are not widely found in diazotrophs, and their functions are yet to be determined. Our results suggested that they play an important role in nitrogenase activity in *Synechocystis* 6803, especially *hesB*, a finding corroborated by studies in the diazotrophic cyanobacterium *Anabaena* sp. PCC7120 (30).

## DISCUSSION

Members of the genus *Cyanothece* that engage in aerobic nitrogen fixation harbor one of the largest *nif* clusters known among cyanobacteria (31). The fact that these unicellular cyanobacteria are preferred by higher photoautotrophs as their nitrogen-fixing partners suggests that their *nif* cluster might confer certain advantages. This hypothesis is further strengthened by recent studies that revealed that close relatives of *Cyanothece* with highly similar *nif* clusters are now functioning as early-stage nitrogen-fixing organelles, called diazoplasts or nitroplasts (1, 4, 32). Therefore, efforts to engineer photoautotrophic systems involving eukaryotic algae, lichens, or plants for the production of nitrogen-rich compounds will benefit from a detailed analysis of the *Cyanothece nif* cluster. In an earlier study, we investigated the efficacy of the *Cyanothece* 51142 *nif* cluster in imparting nitrogen-fixing ability to non-diazotrophic photoautotrophs by introducing it into *Synechocystis* 6803. The engineered *Synechocystis* strain exhibited significant rates of nitrogen fixation, laying the foundation for the current study, which further investigates the functioning of the cluster in photoautotrophs. We focused on identifying a set of essential genes and the optimal expression ratios of some of the key genes involved in the process of nitrogen fixation as the next step toward designing an efficient synthetic cluster for nitrogenase. We successfully re-organized the genes of this cluster to form independent operons in *Synechocystis* 6803 and demonstrated significant nitrogenase activity. Although the highest nitrogenase activity was observed in strain RC5, strain RC4 harboring only 12 *nif* genes also fixed nitrogen at low levels. In a study that introduced the *nif* gene cluster from *Leptolyngbya boryana* into *Synechocystis* 6803, nitrogenase activity around 4 nmol ethylene per mg cell dry weight per hour was demonstrated (33). We determined that under the testing conditions, the ratio of total protein per cell dry weight is 0.25 (data not shown), which translates into an activity of about 1 nmol ethylene per mg total protein in 1 h, a rate very close to that observed in the RC4 strain. Although our engineered RC5 strain exhibits significantly lower activities (45 nmol per mg total protein in 1 h) compared to *Cyanothece* 51142 (1262 nmol per mg total protein in 1 h), the activities are significantly higher compared to the *Synechocystis* 6803 strain expressing genes from *Leptolyngbya boryana* (1 nmol per mg total protein in 1 h) (33). Thus, the nitrogen fixation rates observed in the *Synechocystis* strain expressing genes from *Cyanothece* 51142 are very encouraging and validate our hypothesis that the *Cyanothece nif* cluster offers an optimal starting point for designing synthetic solar-powered nitrogen fixing systems.

In order to convert N_2_ into ammonia, reducing power is required by the nitrogenase enzyme complex. Ferredoxins have been identified as one of the primary electron donors to the connected Fe protein of nitrogenase (34). However, in strain RC5, none of the ferredoxins from *Cyanothece* 51142 were transferred into *Synechocystis* 6803, which indicates that the endogenous ferredoxins in *Synechocystis* 6803 recognize the introduced nitrogenase Fe protein and function as the electron bridge for dinitrogen gas reduction. It has not been determined which ferredoxin gene is essential for electron transfer to nitrogenase in *Cyanothece* 51142; therefore, all three annotated genes in the extended cluster were tested in the RC5 strain. Our results indicated that only the *fdxN* gene positively impacted nitrogenase activity when expressed at a higher level, suggesting that FdxN could be involved in electron transfer to nitrogenase. Studies have revealed that pathways of electron transfer to nitrogenase in metabolically diverse strains differ, and the oxygen dynamics of the cell is an important factor that determines the ferredoxins involved in the process (34). In our study, FdxH and FdxB negatively impacted nitrogenase activity, implying that these ferredoxins could be involved in diverting electrons from nitrogen fixation to other processes in *Synechocystis* 6803, where the cellular oxygen dynamics are very different from *Cyanothece* 51142. It has been shown that electron transport chains from plant organelles function to support nitrogenase activity in engineered *E. coli* (23). The fact that the RC5 strain fixes nitrogen without any ferredoxins from *Cyanothece* suggests that transferring *nif* genes without the ferredoxins from the native cluster might endow higher phototrophs with nitrogenase activity, minimizing the number of genes to be transferred.

In this work, we have described the minimal set of *nif* genes essential for nitrogenase activity in a nondiazotrophic cyanobacterium. However, the complete cellular machinery needed for robust and efficient nitrogen fixation under aerobic conditions, a key piece of the puzzle for engineering photoautotrophs, remains largely unknown. More recently, a transposon sequencing (Tn-Seq) strategy has been used in various non-diazotrophic species, including cyanobacteria, to identify essential gene sets under special conditions (35, 36). Extending such studies to diazotrophic cyanobacterial strains can reveal a more complete picture of the entire cellular machinery needed for high levels of nitrogen fixation.

## MATERIALS AND METHODS

### Microorganisms, culture conditions, and media

All cyanobacterial strains were cultured in 100 mL flasks with fresh BG11 medium and appropriate antibiotics (20 µg/mL kanamycin, 3 µg/mL gentamycin, or 20 µg/mL spectinomycin). Cells were pre-cultured at 30°C under 50 µmol photons⋅m^−2^⋅s^−1^ constant light, with shaking at 150 rpm. For nitrogenase assays, pre-cultured cells were collected and washed with fresh BG11 medium without nitrate (BG11_0_) and resuspended in 500 mL fresh BG11_0_ medium. Cells were grown at 30°C with air bubbling under 12 h light/dark conditions under 50 µmol photons⋅m^−2^⋅s^−1^ of light. All cloning was performed in *E. coli* strain XL1-Blue grown in LB medium in culture tubes or on agar plates at 37°C and supplemented with 50 µg/mL kanamycin, 20 µg/mL gentamycin, or 30 µg/mL spectinomycin, as needed.

### Construction of recombinant plasmids and engineered strains

Plasmids and strains used in this study are listed in Table S1, and all primers are listed in Table S2. Plasmids were constructed by the Gibson isothermal DNA assembly method (37) using linear fragments purified from PCR products. Genomic DNA from the strain C*yanothece* 51142 and *Synechocystis* 6803 were used as templates for PCR. The genetic elements, including promoters, ribosome binding sites, and transcriptional terminators, were chosen from our previous study (17). The plasmid pUC118 (38) was used as the backbone to construct the plasmids used in the promoter assay for structural genes. The broad host plasmid pRSF1010 (39) was used as the backbone for the promoter—EYFP assays—and for the evaluation of the ferredoxin coding genes. The shuttle vector described in our previous study, pCB-SC101, was used for the re-construction work using the bottom–up strategy (17). The plasmid pSL2680 constructed in our lab (18) was used for the knock-out experiments.

Plasmids based on pUC118 and pCB-SC101 vectors were all transformed by natural transformation (40) into the *Synechocystis* 6803 TSyNif-2 and wild-type strains, respectively. A tri-parental conjugation method was used to transfer all pRSF1010 derivative plasmids into *Synechocystis* 6803 cells (41) using a helper strain of *E. coli* containing the pRL443 and pRL623 plasmids (42). Transformants were selected on BG11 agar plates containing 20 µg/mL kanamycin, 3 µg/mL gentamycin, or 20 µg/mL spectinomycin, as needed. Isolated *Synechocystis* 6803 transformants were checked by PCR to confirm the presence of the desired constructs.

All PCR amplifications were performed using Phusion high-fidelity DNA polymerase (Thermo Scientific). Plasmids and PCR products were purified using the GeneJET (Thermo Scientific) plasmid miniprep and gel extraction kits, respectively. Oligonucleotides were designed using SnapGene software (GSL Biotech LLC) and synthesized by IDT (Coralville, IA). All of the plasmids constructed in this study were checked by sequencing to confirm the correct DNA coding information (Genewiz, NJ).

### Fluorescence measurements

Fluorescence intensities were measured as described earlier (17). Briefly, all cultures were adjusted to similar cell densities, with an OD_730 nm_ of 0.2 (about 1 × 10^8^ cells/mL) at the start of the experiment. Three independent replicates of each culture were then transferred to six-well plates and grown for 3 days, followed by fluorescence measurements. The fluorescence intensity and the optical density of each culture were determined on a BioTek Synergy Mx plate reader (BioTek, Winooski, VT). The excitation and emission wavelengths were set to 485 and 528 nm for EYFP, respectively. All fluorescence data were normalized by culture density.

### Measurement of nitrogen fixation activity

Nitrogen fixation activity was measured by an acetylene reduction assay modified from a previously published method (43). The assay was performed as follows: 25 mL of cyanobacterial culture grown in BG11_0_ medium was transferred to a 125 mL air-tight glass vial; 10 µM DCMU was added to the culture; vials were flushed with pure argon; and cultures were incubated in 12 h light/dark conditions. Cells in the sealed vials were cultured overnight, and, at the time point D1, 5 mL acetylene was added into the sealed vials, followed by 3 h of incubation in light at 30°C. 200 µL of gas was sampled from the headspace and injected into an Agilent 6890N gas chromatograph equipped with a Poropak N column and a flame ionization detector using argon as the carrier gas. The temperature of the detector, injector, and oven were 200, 150, and 100°C, respectively.

### Determination of cellular total proteins and cell dry weight

Total protein levels were determined on a plate reader (Bio-Tek Instruments, Winooski, VT) using a bicinchoninic acid assay kit (Pierce, Rockford, IL) according to the manufacturer’s instructions. Cell dry weight was determined from cell pellets of 100 mL culture aliquots that were centrifuged for 15 min at 4°C and 9,000 × *g*, washed twice with distilled water, and dried at 85°C until the weight was constant.

## SUPPLEMENTAL MATERIAL

Fig. S1 mbio.04052-24-s0001.pdf Plasmids for promoter strength assay using EYFP.

Fig. S2 mbio.04052-24-s0002.pdf Plasmid pRC5.

Fig. S3 mbio.04052-24-s0003.pdf pSL2680-based editing plasmids to seamlessly delete target genes in strain RC5.

Supplemental tables mbio.04052-24-s0004.pdf Tables S1 and S2.

ASM does not own the copyrights to Supplemental Material that may be linked to, or accessed through, an article. The authors have granted ASM a non-exclusive, world-wide license to publish the Supplemental Material files. Please contact the corresponding author directly for reuse.
