# Supplementary material for: Investigation of the Cyanothece nitrogenase cluster in Synechocystis: a blueprint for engineering nitrogen-fixing photoautotrophs
Source: mBio. 2025 Feb 25;16(4):e04052-24. doi: 10.1128/mbio.04052-24 (PMC11980358; doi:10.1128/mbio.04052-24)
Supplement: Fig. S1 — Plasmids for promoter strength assay using EYFP. [file mbio.04052-24-s0001.pdf]

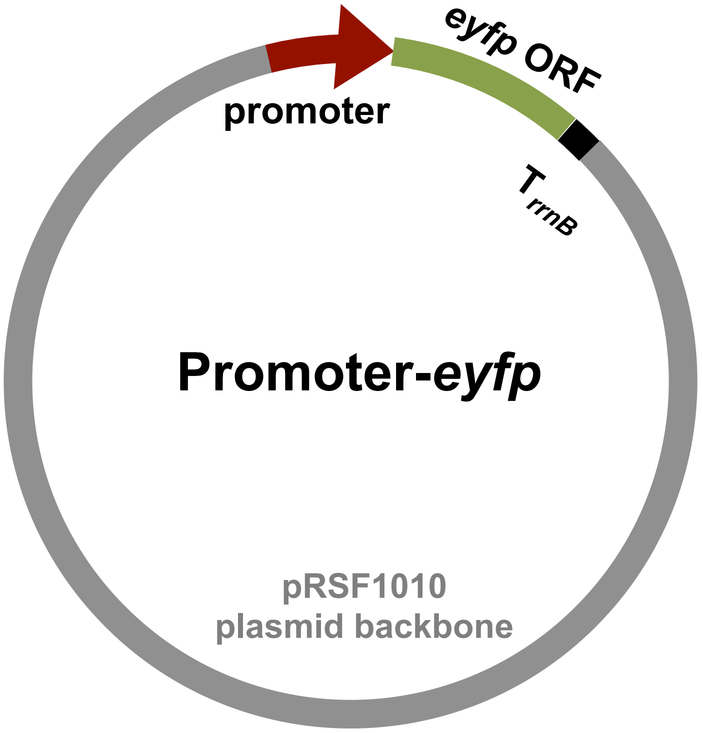

Fig S1

Scheme showing the plasmids for promoter strength assay using EYFP. The plasmids were constructed based on the pRSF1010 backbone. The expression cassette "promoter-*eyfp*-T_{rrnB}" was inserted into the backbone using the Gibson assembly method.
