# Supplementary material for: Investigation of the Cyanothece nitrogenase cluster in Synechocystis: a blueprint for engineering nitrogen-fixing photoautotrophs
Source: mBio. 2025 Feb 25;16(4):e04052-24. doi: 10.1128/mbio.04052-24 (PMC11980358; doi:10.1128/mbio.04052-24)
Supplement: Fig. S2 — Plasmid pRC5. [file mbio.04052-24-s0002.pdf]

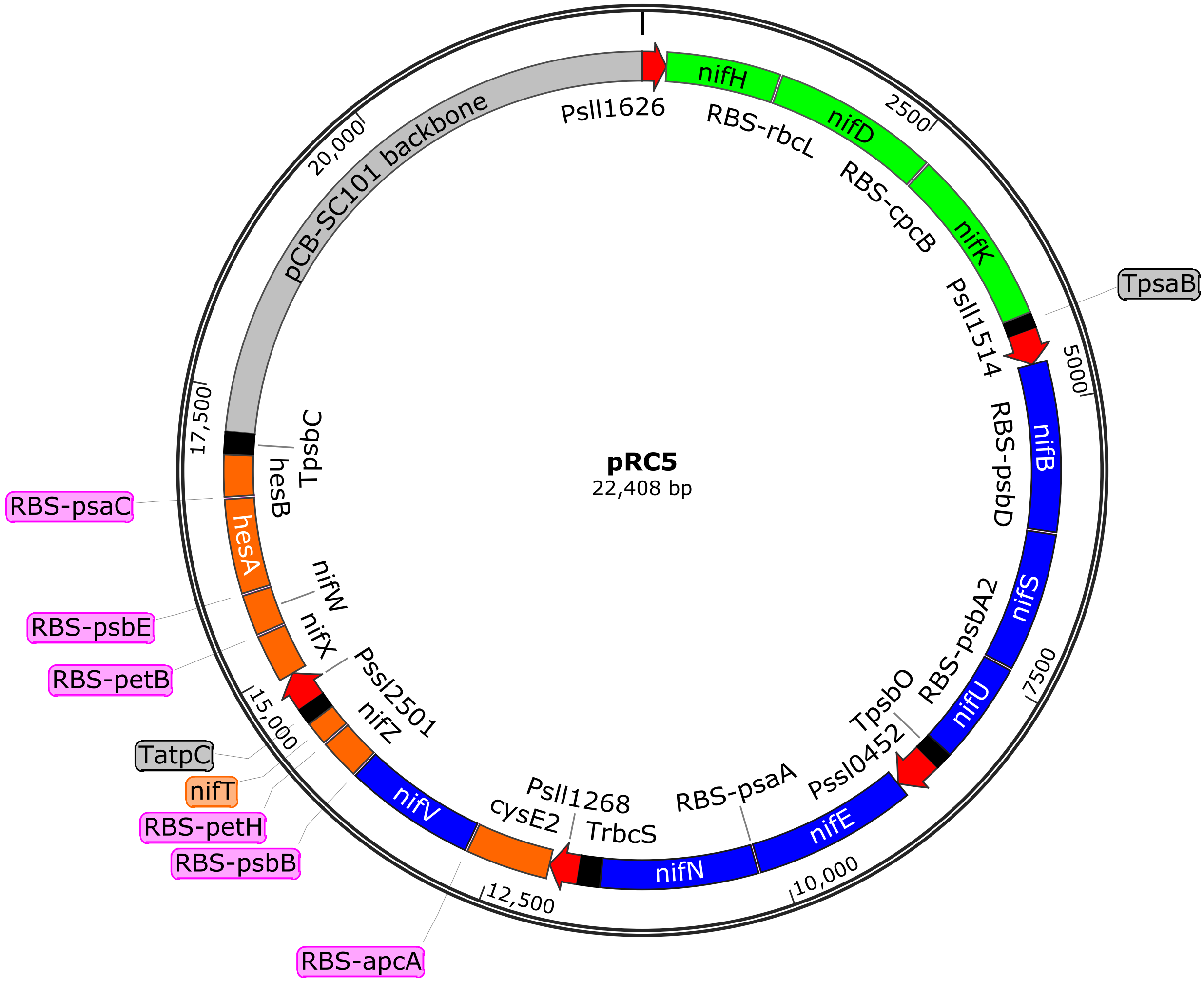

Fig S2

The schematic map of plasmid pRC5. The plasmid contains sixteen re-organized nitrogen fixation genes, *nifHDKBSUENPVZTXWhesAB*. The components of the plasmid are labeled: pCB-SC101 backbone (gray), promoters (red), ribosome binding sites (RBS, pink), transcription terminators (black), and the nitrogen fixation genes (the same colors as in Fig. 2). The sequences of promoters, RBS, terminators, and pCB-SC101 backbone are described in our previous study (1).
