# Supplementary material for: Investigation of the Cyanothece nitrogenase cluster in Synechocystis: a blueprint for engineering nitrogen-fixing photoautotrophs
Source: mBio. 2025 Feb 25;16(4):e04052-24. doi: 10.1128/mbio.04052-24 (PMC11980358; doi:10.1128/mbio.04052-24)
Supplement: Fig. S3 — pSL2680-based editing plasmids to seamlessly delete target genes in strain RC5. [file mbio.04052-24-s0003.pdf]

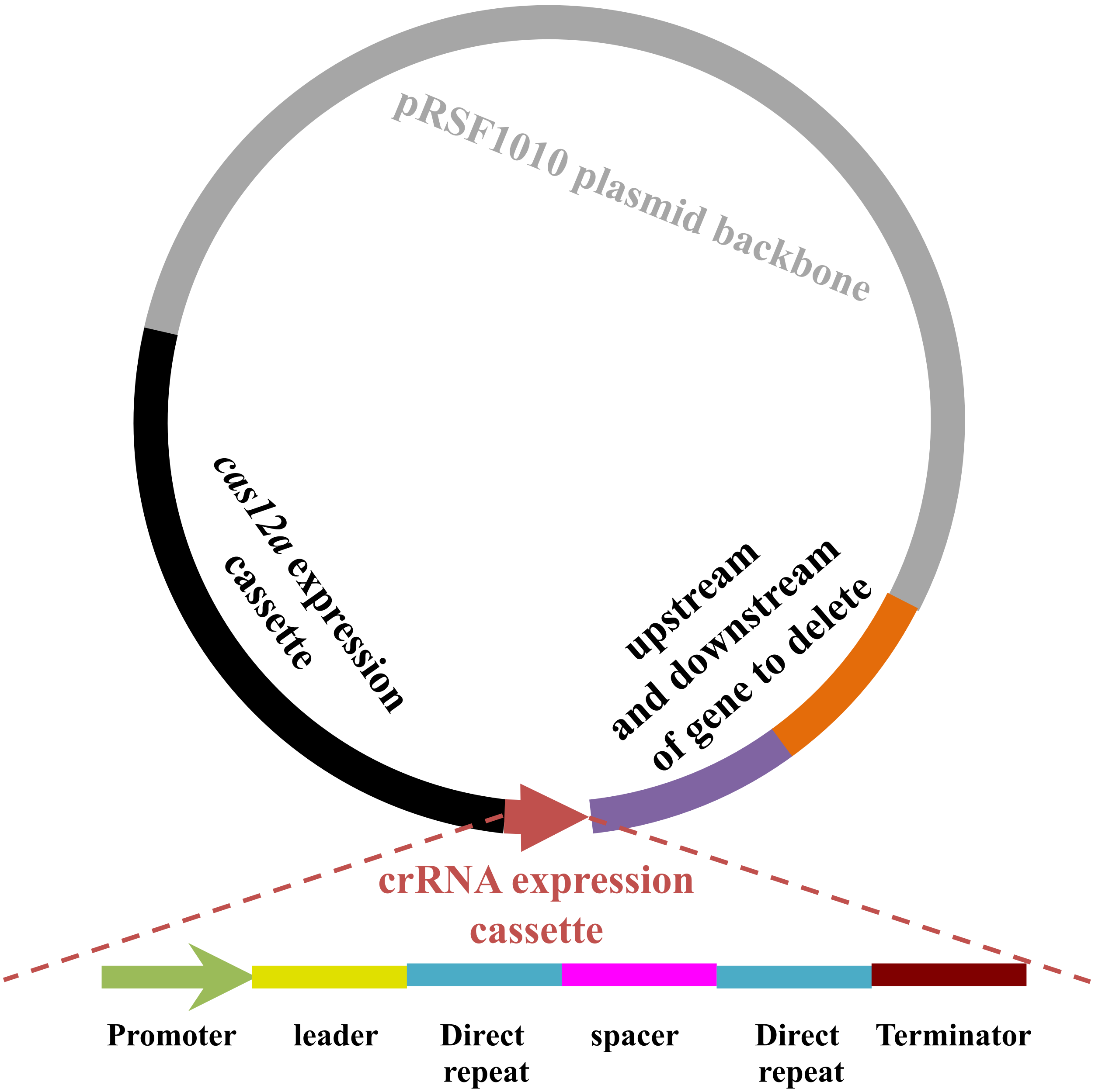

Fig S3
A scheme showing the pSL2680 based editing plasmids to seamlessly delete target genes in strain RC5.
