# Supplementary material for: Investigation of the Cyanothece nitrogenase cluster in Synechocystis: a blueprint for engineering nitrogen-fixing photoautotrophs
Source: mBio. 2025 Feb 25;16(4):e04052-24. doi: 10.1128/mbio.04052-24 (PMC11980358; doi:10.1128/mbio.04052-24)
Supplement: Supplemental tables — Tables S1 and S2. [file mbio.04052-24-s0004.pdf]

Table S1 Strains and plasmids used in this study

Strains and plasmids	Description	Source
**Strain**		
E. coli strain XL1-Blue	Used for DNA cloning	Our lab
Cyanothece sp. ATCC 51142	wild-type strain	Our lab
Synechocystis sp. PCC 6803	wild-type strain	Our lab
TSyNif-2	*Synechocystis* 6803 containing the plasmid pSyNif-2	(1)
TSyNif-14	T2397 with $P_{ssl0452}$ before gene *nifH*	This study
TSyNif-15	T2397 with $P_{sll1626}$ before gene *nifH*	This study
TSyNif-16	T2397 with P_{trc1O} before gene *nifH*	This study
TSyNif-17 (CK)	*Synechocystis* 6803 containing the plasmid pSB-SC101	This study
TSyNif-18 (RC1)	*Synechocystis* 6803 containing the plasmid pSyNif-18	This study
TSyNif-19 (RC2)	*Synechocystis* 6803 containing the plasmid pSyNif-19	This study
TSyNif-20 (RC3)	*Synechocystis* 6803 containing the plasmid pSyNif-20	This study
TSyNif-21 (RC4)	*Synechocystis* 6803 containing the plasmid pSyNif-21	This study
TSyNif-22 (RC5)	*Synechocystis* 6803 containing the plasmid pSyNif-22	This study
TSyNif-23	RC5 containing cassette $P_{slr0701}$-*fdxN*-T_{rrnB} on pRSF1010	This study
TSyNif-24	RC5 containing cassette $P_{ssr2227}$-*fdxN*-T_{rrnB} on pRSF1010	This study
TSyNif-25	RC5 containing cassette P_{psbA2}-*fdxN*-T_{rrnB} on pRSF1010	This study
TSyNif-26	RC5 containing cassette $P_{slr0701}$-*fdxH*-T_{rrnB} on pRSF1010	This study
TSyNif-27	RC5 containing cassette $P_{ssr2227}$-*fdxH*-T_{rrnB} on pRSF1010	This study
TSyNif-28	RC5 containing cassette P_{psbA2}-*fdxH*-T_{rrnB} on pRSF1010	This study
TSyNif-29	RC5 containing cassette P_{trc1O}-*fdxH*-T_{rrnB} on pRSF1010	This study
TSyNif-30	RC5 containing cassette $P_{slr0701}$-*fdxB*-T_{rrnB} on pRSF1010	This study
TSyNif-31	RC5 containing cassette $P_{ssr2227}$-*fdxB*-T_{rrnB} on pRSF1010	This study
TSyNif-32	RC5 containing cassette P_{psbA2}-*fdxB*-T_{rrnB} on pRSF1010	This study
TSyNif-33	RC5 without gene *nifB*	This study
TSyNif-34	RC5 without gene *nifS*	This study
TSyNif-35	RC5 without gene *nifU*	This study
TSyNif-36	RC5 without gene *nifE*	This study
TSyNif-37	RC5 without gene *nifN*	This study
TSyNif-38	RC5 without gene *nifP*	This study
TSyNif-39	RC5 without gene *nifV*	This study
TSyNif-40	RC5 without gene *nifZ*	This study
TSyNif-41	RC5 without gene *nifT*	This study
TSyNif-42	RC5 without gene *nifX*	This study
TSyNif-43	RC5 without gene *nifW*	This study
TSyNif-44	RC5 without gene *hesA*	This study
TSyNif-45	RC5 without gene *hesB*	This study
**Plasmid**		
pRL443	Used for conjugation	(2)
pRL663	Used for conjugation	(2)
pRSF1010	Broad-host-range shuttle vector	(3)
pUC118	DNA cloning vector	(4)
pCB-SC101	Shuttle vector	(5)

Continued

pSL2680	Cpf1-based gene editing plasmid, derived from pRSF1010	(6)
pSL2743	Containing cassette P_{nifH}-*eyfp*-T_{rrnB} on pRSF1010	This study
pSL2745	Containing cassette $P_{ssl0452}$-*eyfp*-T_{rrnB} on pRSF1010	(5)
pSL2751	Containing cassette $P_{sll1626}$-*eyfp*-T_{rrnB} on pRSF1010	(5)
pSL3105	Containing cassette P_{trc1O}-*eyfp*-T_{rrnB} on pRSF1010	(5)
pSyNif-2	Natural *nif* cluster with 24 genes from *Cyanothece* 51142	(1)
pSyNif-18 (pRC1)	Containing re-organized *nifHDK*	This study
pSyNif-19 (pRC2)	Containing re-organized *nifHDKBSU*	This study
pSyNif-20 (pRC3)	Containing re-organized *nifHDKBSUEN*	This study
pSyNif-21 (pRC4)	Containing re-organized *nifHDKBSUENPVZT*	This study
pSyNif-22 (pRC5)	Containing re-organized *nifHDKBSUENPVZTXWhesAB*	This study
pSyNif-23	Containing cassette $P_{slr0701}$-*fdxN*-T_{rrnB} on pRSF1010	This study
pSyNif-24	Containing cassette $P_{ssr2227}$-*fdxN*-T_{rrnB} on pRSF1010	This study
pSyNif-25	Containing cassette P_{psbA2}-*fdxN*-T_{rrnB} on pRSF1010	This study
pSyNif-26	Containing cassette $P_{slr0701}$-*fdxH*-T_{rrnB} on pRSF1010	This study
pSyNif-27	Containing cassette $P_{ssr2227}$-*fdxH*-T_{rrnB} on pRSF1010	This study
pSyNif-28	Containing cassette P_{psbA2}-*fdxH*-T_{rrnB} on pRSF1010	This study
pSyNif-29	Containing cassette P_{trc1O}-*fdxH*-T_{rrnB} on pRSF1010	This study
pSyNif-30	Containing cassette $P_{slr0701}$-*fdxB*-T_{rrnB} on pRSF1010	This study
pSyNif-31	Containing cassette $P_{ssr2227}$-*fdxB*-T_{rrnB} on pRSF1010	This study
pSyNif-32	Containing cassette P_{psbA2}-*fdxB*-T_{rrnB} on pRSF1010	This study
pSyNif-33	pSL2680 based plasmid to knock out *nifB*	This study
pSyNif-34	pSL2680 based plasmid to knock out *nifS*	This study
pSyNif-35	pSL2680 based plasmid to knock out *nifU*	This study
pSyNif-36	pSL2680 based plasmid to knock out *nifE*	This study
pSyNif-37	pSL2680 based plasmid to knock out *nifN*	This study
pSyNif-38	pSL2680 based plasmid to knock out *nifP*	This study
pSyNif-39	pSL2680 based plasmid to knock out *nifV*	This study
pSyNif-40	pSL2680 based plasmid to knock out *nifZ*	This study
pSyNif-41	pSL2680 based plasmid to knock out *nifT*	This study
pSyNif-42	pSL2680 based plasmid to knock out *nifX*	This study
pSyNif-43	pSL2680 based plasmid to knock out *nifW*	This study
pSyNif-44	pSL2680 based plasmid to knock out *hesA*	This study
pSyNif-45	pSL2680 based plasmid to knock out *hesB*	This study

Table S2 Primers used in this study

Primer	Sequence (5′-3′)	Purpose
Testing_PnifH_F	GCTTTCCTGGCTTTGCTTCCCGGGTTTCCCCAGAATTAAC	For promoter strength test
Testing_PnifH_R	TCCTCGCCCTTGCTCACCATTAGAACCCTCGAACGCTACG	
Testing_EYFP_F	ATGGTGAGCAAGGGCGAGGAG	
Testing_TrrnB_R	TACGCTGACTTGACGGGACACGGTTTTAAAGAAAAAGGGCAG	
PHDKchange_US_F	ACAGGGCGCGTACTATGGTTGCTCATGTGGGAATATACAGAC	For promoter switch before *nifHDK*
PHDKchange_US_R	TTATTTGATGCCTGGTCAGTGTTCAGTGATTGTCTC	
PHDKchange_Spe_F	ATCACTGAACACTGACCAGGCATCAAATAAAACGAAAG	
PHDKchange_Spe_R	AATTGCCAAAACCCATGGTACCGCTCTGCCAG	
PHDKchange_TrbcS_F	AGCGGTACCATGGGTTTTGGCAATTACTAAAAAACTG	
PHDKchange_TrbcS_R	TAATTGACAATTGACAATTCCCCAC	
PHDKchange_DS_F	ATGGGACGCAACTCCCAAGG	
PHDKchange_DS_R	CCTGGCCTTTTGCTGGCCTTCAGTCTAAGCTTTGGTAGCAGC	
PHDKchange_Psll1626_F2	TCTAAGTGGGGAATTGTCAATTGTCAATTATCTTCGGC	
PHDKchange_Psll1626_F1	GTCAATTGTCAATTATCTTCGGCGATAACAGTAAC	
PHDKchange_Psll1626_R1	GGAGTTGCGTCCCATGTAATATCTCCTATAGGAATG	
PHDKchange_Psll1626_R2	GGTTAGGAAACCTTGGGAGTTGCGTCCCATGTAATATC	
PHDKchange_PbnlA_F2	TCTAAGTGGGGAATTGTCAATTGTCAATTACAATGAC	
PHDKchange_PnblA_F1	GTCAATTGTCAATTACAATGACCCAATAACTCGTAC	
PHDKchange_PnblA_R1	GGAGTTGCGTCCCATAGCTGTTGCCCTCCAAGGCG	
PHDKchange_PnblA_R2	GGTTAGGAAACCTTGGGAGTTGCGTCCCATAGCTGTTG	
PHDKchange_Ptrc_F2	TCTAAGTGGGGAATTGTCAATTGTCAATTAGACGTC	
PHDKchange_Ptrc_F1	GTCAATTGTCAATTAGACGTCTAAGAAACCATTATTATC	
PHDKchange_Ptrc_R1	GGAGTTGCGTCCCATCTAGTATTTCTCCTCTTTTCTAG	
PHDKchange_Ptrc_R2	GGTTAGGAAACCTTGGGAGTTGCGTCCCATCTAGTATTTC	
BKpCBpSC101_SC101_R	AAGGATCAGCACGTGTAACTGTCAGACCAAGTTTACG	For reconstruction work
BKpCBpSC101_Spe_F	TGACAGTTACACGTGCTGATCCTTCAACTCAGCAAAAG	
RF2rbs_nifD_F	TGTTTATGGAGGACTGACCTAGATGGCAACAGTTGAAGACAATAAG	
RF2rbs_nifD_R	TGAATTAATCTCCTACTTGACTTTATTTATTCCAAGGGGTGCC	
RF2rbs_nifH_R	CTAGGTCAGTCCTCCATAAACACTAAGCTTTGGTAGCAGCTTTATC	
RF2rbs_nifK_F	AGTCAAGTAGGAGATTAATTCAATGGCTCAAAACGTAAACAATATTAAG	
RF2rbs_nifK_R	TAACAGAGGATTAACGAACTAAGTCAAAAGAAATATCC	
RF2rbs_Psll1626_F1	GTCTGACAGTTACACTCTTCGGCGATAACAGTAAC	
RF2rbs_Psll1626_F2	GCGATCGTAAACTTGGTCTGACAGTTACACTCTTCG	
RF2rbs_TpsaB_F	CTTTTGACTTAGTTCGTTAATCCTCTGTTAGGTAATTAAGC	
RF2rbs_TpsaB_R	AGTTGAAGGATCAGCACGTGGCAATGGGGTAAAAATAACAAAAAAC	
RF2rbs_Ttotal_R	CGAACTTTTGCTGAGTTGAAGGATCAGCACGTG	
RF2rbs_Psll1514_F2	AGTTTTTTGTTATTTTTACCCCATTGCCACGTAAAG	
RF2rbs_Psll1514_F1	TTACCCCATTGCCACGTAAAGGTCTCCATTCCTATG	
RF_Psll1514_R	AGACCTGTAGATTGTAACATAATGTTAACTCCTGATGTGTG	
RF2rbs_nifB_F	GTTAACATTATGTTACAATCTACAGGTCTC	
RF2rbs_nifB_R	AAATGCAAATCCTCTTGCGTAGTTAGGCTCCTAATGCTTGTTTTTG	
RF2rbs_nifS_F	CTACGCAAGAGGATTTGCATTTATGAGAGACTGTATATATCTAGAC	
RF2rbs_nifS_R	TTGGTTATAATTCCTTATGTATCTACTTAGCTAAAGTTGCTTTTTC	
RF2rbs_nifU_F	ATACATAAGGAATTATAACCAAATGTGGGAATATACAGACAAAG	
RF2rbs_nifU_R	CAACAAAAAAATTACACTGCAATGACAGTTAATTC	
RF2rbs_TpsbO_F	AACTGTCATTGCAGTGTAATTTTTTTGTTGTGGTAGCTGAC	
RF2rbs_TpsbO_R	AGTTGAAGGATCAGCACGTGCAACAAAACGACAAGAAAATAC	
RF2rbs_Pssl0452_F2	TTTAAGTATTTTCTTGTCGTTTTGTTGCACCAATGAC	
RF2rbs_Pssl0452_F1	GTCGTTTTGTTGCACCAATGACCCAATAACTCGTAC	
RF2rbs_Pssl0452_R	CTTCGTTAGTTTCATAGCTGTTGCCCTCCAAGGCGAC	
RF2rbs_nifE_F	TGGAGGGCAACAGCTATGAAACTAACGAAGAGCAAAATTAAC	
RF2rbs_nifE_R	GCAGGGTTCTCCTCGCTCGACATTACTCGCCTTCTGCTTCTAAG	
RF2rbs_nifN_F	TGTCGAGCGAGGAGAACCCTGCATGACCACTATCTTAAACCCC	
RF2rbs_TrbcS_R	AGTTGAAGGATCAGCACGTGTAATTGACAATTGACAATTCCCC	
RF2rbs_Psll1268_F2	AAGTGGGGAATTGTCAATTGTCAATTACACAGCTAAC	
RF2rbs_Psll1268_F1	AATTGTCAATTACACAGCTAACCCCATTTGATTAGC	

RF2rbs_Psll1268_R	AGTGATCCAAACATTTAAAAGCAACCAGCAGGTG	
RF2rbs_nifP_F	CTGGTTGCTTTTAAATGTTTGGATCACTCCCTGTC	
RF2rbs_nifP_R	GGATGGATTCCTCCGTAAAGATTCAAGGTAGTTTTTGTATCTTTTG	
RF2rbs_nifV_F	ATCTTTACGGAGGAATCCATCCATGAATCATGTCCATGTTAATGATAC	
RF2rbs_nifV_R	TGACGCTCCTTCTAGTAACGAATTATACAATCCCATGAGACATAC	
RF2rbs_nifZ_F	TTCGTTACTAGAAGGAGCGTCAATGGGATTGTATAATCCAGGTG	
RF2rbs_nifZ_R	GGGTTAATTGCTCCCTACTCAATTAAACCTGAGACTCTTGTAGAG	
RF2rbs_nifT_F	TTGAGTAGGGAGCAATTAACCCATGAAAGTGATCTTAAGCAGAG	
RF2rbs_nifT_R	ATCACTAAATGGGCTAGAGACGTTTTGCGTCCAAG	
RF2rbs_TatpC_F	AAAACGTCTCTAGCCCATTTAGTGATCTTTTCGTTG	
RF2rbs_TatpC_R	AGTTGAAGGATCAGCACGTGTCGGCGCTTGGTCAAATCTC	
RF2rbs_Pssl2501_F2	TTTGTTGAGATTTGACCAAGCGCCGACACCAGAAAAC	For reconstruction work
RF2rbs_Pssl2501_F1	CCAAGCGCCGACACCAGAAAACTAATCTGCCCTTTG	
RF2rbs_Pssl2501_R	AAATGCAACTTTCATAGGGCTAAAACCGCTAATATC	
RF2rbs_nifX_F	TTTTAGCCCTATGAAAGTTGCATTTACTACTAG	
RF2rbs_nifX_R	TGAAGCTAGACTTCTCTTAAGGTTATTCATCCTCTGCTAAAAAGTTAGG	
RF2rbs_nifW_F	CCTTAAGAGAAGTCTAGCTTCAATGACTATTACTGCTGATACCC	
RF2rbs_nifW_R	TTTGTGTTCTCCTTCAATTTTTCTATTGCATAATGCCAGCTTC	
RF2rbs_hesA_F	AAAAATTGAAGGAGAACACAAAATGAACTTCACCCCTACTGAAC	
RF2rbs_hesA_R	TGACTATCGGCTCCTTAACCGATTAACTCCTGACTCCTGACTC	
RF2rbs_hesB_F	TCGGTTAAGGAGCCGATAGTCAATGACTGTCACTCTTACTGAAAAAG	
RF2rbs_hesB_R	AAAAGTCTCAATTTAGCTACAAGAAGTAGCTTG	
RF2rbs_TpsbC_F	TTCTTGTAGCTAAATTGAGACTTTTCTGATTTTGC	
RF2rbs_TpsbC_R	GCTGAGTTGAAGGATCAGCACGTGCGGGGAAAGGTTTTTGGAAGC	
RF3fdx-P0701_F	TTCCTGGCTTTGCTTCCCACCGAGAACTAAGACAAAAATTAC	
RF3fdx_TapcC_R	TTACGCTGACTTGACGGGACACTCAGGCGATCGCCGATGCGC	
RF3fdx_fdxN_F	ATGAGTTACACTATCACCAATGAATG	
RF3fdx_P2227_F	CTTTCCTGGCTTTGCTTCCCACAGTGAATAAAGTTAAATATTGCTTGCC	For *fdxNHB* genes expression
RF3fdx_P2227_R	CATTGGTGATAGTGTAACTCATTGATAATTTATTTCTCGTAGGTCTAAC	
RF3fdx_PpsbA2_F	CTTTCCTGGCTTTGCTTCCCACCTCCATTGTCCCTGAAAATCAG	
RF3fdx_PpsbA2_R	CATTGGTGATAGTGTAACTCATTTGGTTATAATTCCTTATGTATTTGTC	
RF3fdx_Ptrc_F	CTTTCCTGGCTTTGCTTCCCACGACGTCTAAGAAACCATTATTATC	
RF3fdx_Ptrc_R	CATTGGTGATAGTGTAACTCATCTAGTATTTCTCCTCTTTTCTAGTATG	
CRISPR-gRNATmpt_F	GAGCCTTTTGTATTAGTAGCCG	
CRISPR-gRNATmpt_R	GTACCAACTACCGCATTAAAGC	
gRNAseq_F	GAAGAGTATTTTGAGTTCGTGC	
CRISPRnifB_gRNA_F1	GCGAACCCCAAACAAACCTTGTCTAAGAACTTTAAATAATTTCTAC	
CRISPRnifB_gRNA_F2	TTTAAATAATTTCTACTGTTGTAGATGCGAACCCCAAACAAACCTTG	
CRISPRnifB_gRNA_R	TTAAGAACTCATCAGGTACCAACTACCGCATTAAAGC	
CRISPRnifB_US_F	TGCGGTAGTTGGTACCTGATGAGTTCTTAATGGGC	
CRISPRnifB_US_R	TATACAGTCTCTCATAATGTTAACTCCTGATGTGTG	
CRISPRnifB_DS_F	TCAGGAGTTAACATTATGAGAGACTGTATATATCTAGAC	
CRISPRnifB_DS_R	TACAGATCCTCTAGAGTCGACGGAGTCCCATAGCTCTTAAGAC	
CRISPRnifS_gRNA_F1	TATGTGCGTAAAGGAACCCGGTCTAAGAACTTTAAATAATTTCTAC	
CRISPRnifS_gRNA_F2	TTTAAATAATTTCTACTGTTGTAGATTATGTGCGTAAAGGAACCCG	For knocking-out genes
CRISPRnifS_gRNA_R	CCAGGACCGGCAATAGTACCAACTACCGCATTAAAGC	
CRISPRnifS_US_F	TGCGGTAGTTGGTACTATTGCCGGTCCTGGTGATC	
CRISPRnifS_US_R	TAATTCCTTATGTATTTAGGCTCCTAATGCTTGTTTTTGC	
CRISPRnifS_DS_F	GCATTAGGAGCCTAAATACATAAGGAATTATAACCAAATG	
CRISPRnifS_DS_R	TACAGATCCTCTAGAGTCGACGAAGACGACCACCTTAACGGC	
CRISPRnifU_gRNA_F1	CCGGAAGAGAAAATGCACTGGTCTAAGAACTTTAAATAATTTCTAC	
CRISPRnifU_gRNA_F2	TTTAAATAATTTCTACTGTTGTAGATCCGGAAGAGAAAATGCACTG	
CRISPRnifU_gRNA_R	TTCGGCACCGAGTAAGTACCAACTACCGCATTAAAGC	
CRISPRnifU_US_F	TGCGGTAGTTGGTACTTACTCGGTGCCGAACCTTC	
CRISPRnifU_US_R	TACCACAACAAAAAACTACTTAGCTAAAGTTGCTTTTTC	
CRISPRnifU_DS_F	ACTTTAGCTAAGTAGTTTTTTGTTGTGGTAGCTGAC	
CRISPRnifU_DS_R	TACAGATCCTCTAGAGTCGACGCTCCAAGGCGACTACCTTTC	
CRISPRnifN_gRNA_F1	GCTGGGTTAGATGCTGTTGAGTCTAAGAACTTTAAATAATTTCTAC	

CRISPRnifN_gRNA_F2	TTTAAATAATTTCTACTGTTGTAGATGCTGGGTTAGATGCTGTTGAG	
CRISPRnifN_gRNA_R	ACACCCAAAGGTTTGGTACCAACTACCGCATTAAAGC	
CRISPRnifN_US_F	TGCGGTAGTTGGTACCAAACCTTTGGGTGTGCTTC	
CRISPRnifN_US_R	CTAACATTGAATTGAATTTAGTCAACATCGGGGAC	
CRISPRnifN_DS_F	CGATGTTGACTAAATTCAATTCAATGTTAGCCCGC	
CRISPRnifN_DS_R	TACAGATCCTCTAGAGTCGACGTTTGCCGTGTTCTAAAGGAC	
CRISPRnifP_gRNA_F1	TCGATCATGGTATGGGGGTGGTCTAAGAACTTTAAATAATTTCTAC	
CRISPRnifP_gRNA_F2	TTAAATAATTTCTACTGTTGTAGATTCGATCATGGTATGGGGGTGG	
CRISPRnifP_gRNA_R	AACCCAGCTAAACGGGTACCAACTACCGCATTAAAGC	
CRISPRnifP_US_F	TGCGGTAGTTGGTACCCGTTTAGCTGGGTTAGATGC	
CRISPRnifP_US_R	ATGGACATGATTCATTTAAAAGCAACCAGCAGGTGG	
CRISPRnifP_DS_F	GCTGGTTGCTTTTAAATGAATCATGTCCATGTTAATG	
CRISPRnifP_DS_R	TACAGATCCTCTAGAGTCGACGCTCATCTGGGGAAAAGGGTTC	
CRISPRnifV_gRNA_F1	ACCACGGCTCAAAAAGTGGGGTCTAAGAACTTTAAATAATTTCTAC	
CRISPRnifV_gRNA_F2	TTAAATAATTTCTACTGTTGTAGATACCACGGCTCAAAAAGTGGGG	
CRISPRnifV_gRNA_R	AAATGGGGTTAGCTGGTACCAACTACCGCATTAAAGC	
CRISPRnifV_US_F	TGCGGTAGTTGGTACCAGCTAACCCCATTTGATTAGC	
CRISPRnifV_US_R	CTAGTAACGAATCAAGGTAGTTTTTGTATCTTTTGTTC	
CRISPRnifV_DS_F	CAAAAACTACCTTGATTCGTTACTAGAAGGAGCGTC	
CRISPRnifV_DS_R	TACAGATCCTCTAGAGTCGACGCGCTAATATCCCTAAACGTTC	
CRISPRnifZ_gRNA_F1	CGGGTAAAGAAGTGGGCGAAGTCTAAGAACTTTAAATAATTTCTAC	
CRISPRnifZ_gRNA_F2	TTAAATAATTTCTACTGTTGTAGATCGGGTAAAGAAGTGGGCGAAG	
CRISPRnifZ_gRNA_R	CCCACTTCCAACTCAGTACCAACTACCGCATTAAAGC	
CRISPRnifZ_US_F	TGCGGTAGTTGGTACTGAGTTGGAAGTGGGTATTCC	
CRISPRnifZ_US_R	TGCTCCCTACTCAATTATACAATCCCATGAGACATACTCC	
CRISPRnifZ_DS_F	ATGGGATTGTATAATTGAGTAGGGAGCAATTAACCCATG	
CRISPRnifZ_DS_R	TACAGATCCTCTAGAGTCGACGGATTAAACGGGCTGCTGCACTG	
CRISPRnifT_gRNA_F1	GAGGAAGCAGTGGTTAACGAGTCTAAGAACTTTAAATAATTTCTAC	
CRISPRnifT_gRNA_F2	TTAAATAATTTCTACTGTTGTAGATGAGGAAGCAGTGGTTAACGAG	For knocking-out genes
CRISPRnifT_gRNA_R	GTTAAAGGGTTAAGAGTACCAACTACCGCATTAAAGC	
CRISPRnifT_US_F	TGCGGTAGTTGGTACTCTTAACCCTTTAACCACGGC	
CRISPRnifT_US_R	AGATCACTAAATGGGTTAAACCTGAGACTCTTGTAGAG	
CRISPRnifT_DS_F	GAGTCTCAGGTTTAACCCATTTAGTGATCTTTTCGTTGC	
CRISPRnifT_DS_R	TACAGATCCTCTAGAGTCGACGTGGGCGATAAGTAAAGAGAAC	
CRISPRnifX_gRNA_F1	CTCTATGTTTCTGCGATCGGGTCTAAGAACTTTAAATAATTTCTAC	
CRISPRnifX_gRNA_F2	TTAAATAATTTCTACTGTTGTAGATCTCTATGTTTCTGCGATCGGG	
CRISPRnifX_gRNA_R	TTTCATTTGTACCGGTACCAACTACCGCATTAAAGC	
CRISPRnifX_US_F	TGCGGTAGTTGGTACCGGTACAAATGAAACGCAGTC	
CRISPRnifX_US_R	AGCAGTAATAGTCATAGGGCTAAAACCGCTAATATCC	
CRISPRnifX_DS_F	AGCGGTTTTAGCCCTATGACTATTACTGCTGATACCC	
CRISPRnifX_DS_R	TACAGATCCTCTAGAGTCGACGATGATATGGACGACGTTTGGC	
CRISPRnifW_gRNA_F1	CTTATCGCCCAAGTCGACAAGTCTAAGAACTTTAAATAATTTCTAC	
CRISPRnifW_gRNA_F2	TTTAAATAATTTCTACTGTTGTAGATCTTATCGCCCAAGTCGACAA	
CRISPRnifW_gRNA_R	GTTAACCACTGCTTCGTACCAACTACCGCATTAAAGC	
CRISPRnifW_US_F	TGCGGTAGTTGGTACGAAGCAGTGGTTAACGAAACC	
CRISPRnifW_US_R	TCTCCTTCAATTTTTTTATTCATCCTCTGCTAAAAAG	
CRISPRnifW_DS_F	GCAGAGGATGAATAAAAAAATTGAAGGAGAACACAAAATG	
CRISPRnifW_DS_R	TACAGATCCTCTAGAGTCGACGATCTTGTTCAAAGCGGACATC	
CRISPRhesA_gRNA_F1	GTACAACAGGCCGATATTGCGTCTAAGAACTTTAAATAATTTCTAC	
CRISPRhesA_gRNA_F2	TTAAATAATTTCTACTGTTGTAGATGTACAACAGGCCGATATTGCG	
CRISPRhesA_gRNA_R	TCTGTTCACTACGGTGTACCAACTACCGCATTAAAGC	
CRISPRhesA_US_F	TGCGGTAGTTGGTACACCGTAGTGAACAGATTGTCC	
CRISPRhesA_US_R	CGGCTCCTTAACCGACTATTGCATAATGCCAGCTTC	
CRISPRhesA_DS_F	GGCATTATGCAATAGTCGGTTAAGGAGCCGATAGTC	
CRISPRhesA_DS_R	TACAGATCCTCTAGAGTCGACGAATGATGTCGTCGTGCACAAC	
CRISPRhesB_gRNA_F1	GTCAAAGAAGCCAACAACGAGTCTAAGAACTTTAAATAATTTCTAC	
CRISPRhesB_gRNA_F2	TTAAATAATTTCTACTGTTGTAGATGTCAAAGAAGCCAACAACGAG	
CRISPRhesB_gRNA_R	GGGGAAGGCTTTGTCGTACCAACTACCGCATTAAAGC	

CRISPRhesB_US_F	TGCGGTAGTTGGTACGACAAAGCCTTCCCCGATCTC	For knocking-out genes
CRISPRhesB_US_R	CAGAAAAGTCTCAATTTAACTCCTGACTCCTGACTCC	
CRISPRhesB_DS_F	GAGTCAGGAGTTAAATTGAGACTTTTCTGATTTTGCAAAG	
CRISPRhesB_DS_R	TACAGATCCTCTAGAGTCGACGTCAAGTATGACGGGCTGATAC	

REFERENCES

1. D. Liu, M. Liberton, J. Yu, H. B. Pakrasi, M. Bhattacharyya-Pakrasi, Engineering nitrogen fixation activity in an oxygenic phototroph. *mBio* 9, (2018).
2. N. F. Tsinoremas, A. K. Kutach, C. A. Strayer, S. S. Golden, Efficient gene transfer in *Synechococcus* sp. strains PCC 7942 and PCC 6301 by interspecies conjugation and chromosomal recombination. *J. Bacteriol.* 176, 6764-6768 (1994).
3. S. S. Golden, J. Brusslan, R. Haselkorn, Genetic engineering of the cyanobacterial chromosome. *Methods Enzymol.* 153, 215-231 (1987).
4. J. Vieira, J. Messing, Production of single-stranded plasmid DNA. *Methods Enzymol.* 153, 3-11 (1987).
5. D. Liu, H. B. Pakrasi, Exploring native genetic elements as plug-in tools for synthetic biology in the cyanobacterium *Synechocystis* sp. PCC 6803. *Microb. Cell Fact.* 17, 48 (2018).
6. J. Ungerer, H. B. Pakrasi, Cpf1 Is a versatile tool for CRISPR genome editing across diverse species of cyanobacteria. *Sci. Rep.* 6, 39681 (2016).
